# A mixed-methods feasibility and external pilot study to inform a large pragmatic randomised controlled trial of the effects of surgical wound dressing strategies on surgical site infections (Bluebelle Phase B): study protocol for a randomised controlled trial

**DOI:** 10.1186/s13063-017-2102-5

**Published:** 2017-08-29

**Authors:** The Bluebelle Study Group, Barnaby C. Reeves, Lazaros Andronis, Jane M. Blazeby, Natalie S. Blencowe, Melanie Calvert, Joanna Coast, Tim Draycott, Jenny L. Donovan, Rachael Gooberman-Hill, Robert J. Longman, Laura Magill, Jonathan M. Mathers, Thomas D. Pinkney, Chris A. Rogers, Leila Rooshenas, Andrew Torrance, Nicky J. Welton, Mark Woodward, Kate Ashton, Katarzyna D. Bera, Gemma L. Clayton, Lucy A. Culliford, Jo C. Dumville, Daisy Elliott, Lucy Ellis, Hannah Gould-Brown, Rhiannon C. Macefield, Christel McMullan, Caroline Pope, Dimitrios Siassakos, Sean Strong, Helen Talbot

**Affiliations:** 1 Health Economics Unit, School of Health and Population Sciences, University of Birmingham, Birmingham, UK; 2 School of Social and Community Medicine, University of Bristol, Bristol, UK; 3 University Hospitals Bristol NHS Foundation Trust, Bristol, UK; 4 Centre for Patient Reported Outcomes Research, University of Birmingham, Birmingham, UK; 5 Institute of Applied Health Research, University of Birmingham, Birmingham, UK; 6 North Bristol NHS Trust, Bristol, UK; 7 NIHR Collaboration for Leadership in Applied Health Research and Care West at University Hospitals Bristol NHS Trust, Bristol, UK; 8 Musculoskeletal Research Unit, School of Clinical Sciences, University of Bristol, Bristol, UK; 9 Academic Department of Surgery, Queen Elizabeth Hospital, University of Birmingham, Birmingham, UK; 10 School of Clinical Sciences, University of Bristol, Bristol, UK; 11 Department of Surgery, Sandwell and West Birmingham NHS Trust, Birmingham, UK; 12 School of Nursing, Midwifery & Social Work, University of Manchester, Manchester, UK

**Keywords:** Pilot study, Feasibility study, Randomised controlled trial, Wound dressing, Abdominal surgery, Caesarean section, Wound dressing, Surgical site infection

## Abstract

**Background:**

Surgical site infections (SSIs) are common, occurring in up to 25% of > 4 million operations performed in England each year. Previous trials of the effect of wound dressings on the risk of developing a SSI are of poor quality and underpowered.

**Methods/Design:**

This study is a feasibility and pilot trial to examine the feasibility of a full trial that will compare simple dressings, no dressing and tissue-glue as a dressing. It is examining the overall acceptability of trial participation, identifying opportunities for refinement, testing the feasibility of and validating new outcome tools to assess SSI, wound management issues and patients’ wound symptom experiences. It is also exploring methods for avoiding performance bias and blinding outcome assessors by testing the feasibility of collecting wound photographs taken in theatre immediately after wound closure and, at 4–8 weeks after surgery, taken by participants themselves or their carers. Finally, it is identifying the main cost drivers for an economic evaluation of dressing types. Integrated qualitative research is exploring acceptability and reasons for non-adherence to allocation. Adults undergoing primary elective or unplanned abdominal general surgery or Caesarean section are eligible. The main exclusion criteria are abdominal or other major surgery less than three months before the index operation or contraindication to dressing allocation. The trial is scheduled to recruit for nine months. The findings will be used to inform the design of a main trial.

**Discussion:**

This pilot trial is the first pragmatic study to randomise participants to no dressing or tissue-glue as a dressing versus a simple dressing. Early evidence from the ongoing pilot shows that recruitment is proceeding well and that the interventions are acceptable to participants. Combined with the qualitative findings, the findings will inform whether a main, large trial is feasible and, if so, how it should be designed.

**Trial registration:**

ISRCTN49328913. Registered on 20 October 2015.

**Electronic supplementary material:**

The online version of this article (doi:10.1186/s13063-017-2102-5) contains supplementary material, which is available to authorized users.

## Background

It has been estimated that over 200 million operations are performed worldwide each year and about 4.5 million in England [1]. At the end of each procedure the skin edges of the wound are approximated using sutures or clips. Closing a surgical incision in this way creates what is called ‘a closed primary wound’. Following most surgery in adults, it is standard practice to cover closed primary wounds with a dressing. However, it is rare to apply dressings to closed primary wounds in children and in some specialist areas of adult surgery [2]. There are many different types of dressing available. They range from simple (basic) to complex (advanced) dressings; the latter may have absorbent or low adherence properties and some may interact with the wound to improve healing. The costs of different dressings vary too, from a few pence for basic wound contact dressings to over £15 for advanced antimicrobial dressings [3].

Evidence about the effects of wound dressings on surgical wound healing in both adult and paediatric practice has been systematically reviewed [4]. The review found no evidence to suggest that covering surgical wounds with dressings reduces the risk of a surgical site infection (SSI) or that one wound dressing is more effective than another in reducing scarring, controlling pain, promoting patient acceptability or ease of dressing removal. However, the review authors also found that the available evidence was of poor quality; most studies were small and judged to be at high risk of bias. They acknowledged the logistical challenge of conducting a sufficiently large randomised controlled trial (RCT) to detect a small target difference in the frequency of SSIs with adequate power; a small target difference is likely to be required because of the high cost of a SSI [5]. The authors suggested that choices between wound dressing strategies (between dressing versus no dressing as well as between different dressings) could be based instead on their effects on dressing costs and patient acceptability issues, such as managing exudate and symptoms.

SSIs complicate up to 25% of surgical procedures [6–8]. Many SSIs resolve with simple antibiotic treatment but the more serious SSIs cause morbidity and pain, discomfort and inconvenience for patients. SSIs are costly for health services; the average cost of a SSI has been estimated to be at least £4600 (lower 95% confidence limit) [5]. After some operations, a SSI can threaten the principal outcome of the operation, the future health of the patient and possibly even their life [9–13]. Therefore, every effort is made before, during and after surgery to minimise the risk of developing a SSI.

A key challenge in designing a RCT to evaluate the effect of an intervention on SSI is choosing a method to assess SSI that is feasible for a large RCT and satisfies a wide range of stakeholders likely to have an interest in the findings. Definitions of a SSI have been described in another review [14], which concluded that SSI definitions varied between surveillance programmes and hospitals and lacked good agreement. The authors recommended that future research should focus on developing a SSI measure with satisfactory psychometric properties; the measure should be formulated with the objective of detecting SSIs that are important to patients and health services, include post-discharge surveillance, and be suitable for application in everyday settings.

The Bluebelle study has been funded by the National Institute for Health Research (NIHR) Health Technology Assessment (HTA) programme with the aim of establishing whether it is possible to carry out a large RCT to compare the effectiveness and cost-effectiveness of wound dressing types or no dressing on participant outcome [15]. It also aims to develop new and better outcome measures for the evaluation of wound dressings to use in a main trial. The Bluebelle study has two parts, Phase A and Phase B. Phase A comprised:

- case studies in general abdominal and obstetric surgery to understand and explore the current use of dressings and views about not using dressings [16];
- a survey of dressings currently used after primary wound closure [17];
- a review of the effectiveness and costs of dressings and contextual information (to update a Cochrane review [4]);
- development and validation of questionnaire tools to assess SSI after discharge [18] and practical wound management and participant symptom experience; [19]
- research to define metrics to measure the quality of wound closure and, through literature review, non-participant observations in theatres and interviews with surgeons;
- several meetings with people who had had surgery during Phase A to consider key aspects of the study and participants’ involvement, including information from participants’ interviews.

Phase B, a feasibility and external pilot trial [20] informed by Phase A, is the subject of this protocol paper.

### Aims and objectives

The overall aim of Phase B of the Bluebelle Study is to carry out a feasibility and external pilot trial to establish whether it is possible to carry out a large definitive RCT. Based on findings from Phase A, the pilot trial is designed to investigate the practicability of an RCT to compare the effectiveness and cost-effectiveness of simple dressings, tissue adhesive used as a dressing (glue-as-a-dressing) and no dressing. The specific objectives are to:

1. Establish the numbers of potentially eligible participants at different hospitals who can be approached about the trial, and the proportions confirmed as eligible, recruited and randomised
2. Pilot the randomisation process and attempt to address any issues before progressing to a main trial, including the risk of performance bias if allocation is revealed before wound closure
3. Assess acceptability of the trial interventions and processes to participants and clinical staff using qualitative research methods, including methods to promote adherence
4. Assess adherence to dressing type allocation and the follow-up protocol and reasons for non-adherence
5. Assess the feasibility of collecting a range of secondary outcomes and resource use
6. Establish the validity and reliability of questionnaire tools for identifying SSI (wound healing questionnaire [WHQ]) and describing wound management (wound management questionnaire [WMQ]) and a participant’s experiences of wound care (wound experience questionnaire [WEQ])
7. Explore the feasibility of obtaining digital photographs of wounds taken by theatre personnel in theatre after wound closure and, at 4–8 weeks after surgery, taken by participants themselves or their carers
8. Explore aspects of the trial design and conduct with a patient and public involvement group to inform the conduct of Phase B and the design of a future main trial, following INVOLVE guidance [21]

## Methods/Design

### Study design

**Fig. 1:**
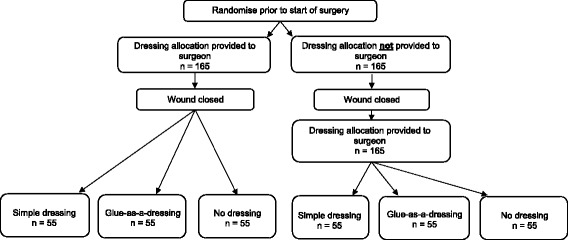
Trial schema. Schema showing the pathway for patients recruited into the Bluebelle Phase B external pilot trial, including the double randomisation

**Fig. 2:**
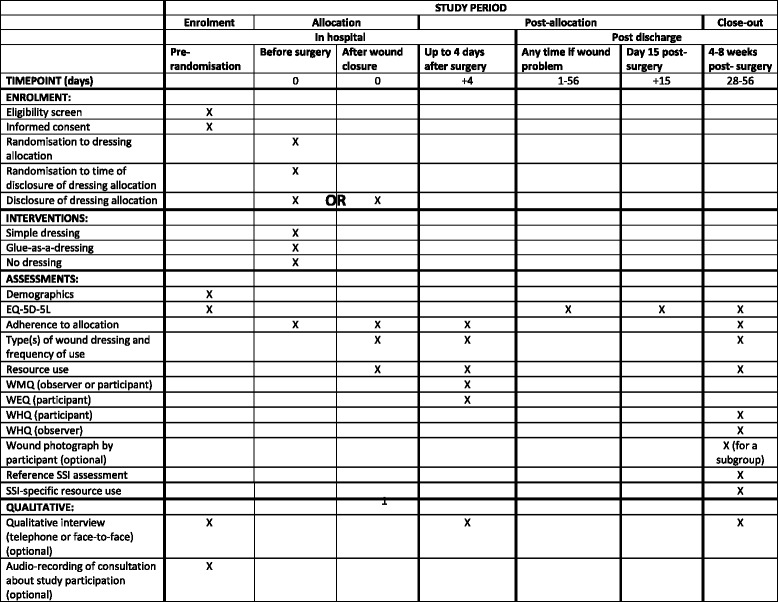
SPIRIT figure. The figure shows the phases of the trial and data collection time points

Phase B of the Bluebelle study is a pragmatic feasibility and pilot three-group parallel RCT (Fig. 1), using both quantitative and qualitative research methods. A SPIRIT Figure shows the different data colelction steps of the pilot trial (Fig. 2); a completed SPIRIT checklist is available as an additional file (Additional file 1: SPIRIT checklist).

### Trial registration, research approvals and research governance

The trial is registered with Current Controlled Trials; ISRCTN49328913 was assigned on 20 October 2015. The sponsor for the trial is University Hospitals Bristol NHS Foundation Trust (research@uhbristol.nhs.uk) and has responsibility for monitoring trial conduct.

The funder and sponsor have no role in: study design; data collection, management or analysis; writing of reports; or any future decisions to submit reports for publication. The funder appointed an independent Study Steering Committee, overseeing both Phase A and Phase B. The funder and the Research Ethics Committee (REC) agreed that a Data Monitoring and Safety Committee was unnecessary. Phase B was coordinated by the Clinical Trials and Evaluation Unit. A Study Executive Group monitors progress approximately monthly.

### Study population

The setting for the research is secondary care, i.e. acute and maternity NHS hospitals. Patients aged 16 years or older undergoing primary elective or unplanned open or laparoscopic abdominal general surgery (including, but not limited to, gastrectomy for benign or malignant disease, cholecystectomy, anti-reflux procedures, hepatic resection, small or large bowel resection for benign or malignant conditions, abdominal wall hernia surgery [inguinal, femoral, incisional, epigastric and para-umbilical]) or elective or unplanned obstetric surgery (Caesarean section) are eligible. Patients undergoing simultaneous abdominal and chest surgery are eligible but only the abdominal wounds are allocated to one of the study interventions. At the time of recruitment, research nurses emphasise the need to attend a follow-up clinic 4–8 weeks after surgery and do not recruit patients who are unable to do so.

Patients meeting the above criteria but with any of the following characteristics are ineligible:

- abdominal or other major surgery less than three months before the index operation;
- the surgeon intends to ‘close’ the wound with tissue adhesive (glue);
- any contraindication to one of the dressing allocations including allergy to dressings;
- surgery where no skin incision occurs;
- lacking capacity to consent;
- inability to complete patient-reported outcome questionnaires;
- detained in the prison service.

All reasons for ineligibility are being recorded in the trial screening log, allowing the trial results to be reported in accordance with the CONSORT guidelines.

Eligible patients are provided with written information (invitation letter and participant information leaflet [PIL] (Additional file 2), reviewed by the REC) and given as long as possible to consider the study before being approached for participation (usually more than 4 h for elective surgery and usually more than 1 h for unplanned surgery). Those having elective surgery may also be given these documents at a clinic visit before the operation or be sent them in advance of admission. Research nurses or surgeons, who are responsible for approaching potential participants, do not request consent if a patient asks for longer thinking time and do not approach any patient who appears visibly distressed.

Participants are also asked to consent to four optional aspects of the trial, designed to explore feasibility (Additional file 3). The first relates to an interview about the acceptability of, and adherence to, the allocated dressing type (objectives 3 and 4). The second allows a local member of the research team to take a photograph of the wound(s) in the operating theatre, immediately after wound closure (objective 2). The third relates to the participant’s willingness to take a photograph of their wound(s) themselves 4–8 weeks after the operation and to send it to the research team (described to participants as a ‘wound selfie’, a term which was readily understood; objective 7). The fourth covers consent for a skin transfer to be applied after the operation to remind staff that the participant is in the study.

Surgery is carried out according to local protocols for the operation. Apposition of wound edges and the method of closure of the skin is at the discretion of the surgeon and may include sutures, clips, wound closure strips or combinations of these wound closure methods.

### Randomisation

Participants are randomised to one of three dressing groups. Participants are also randomised to disclosure of the dressing group allocation to the surgeon before wound closure or after wound closure. These two randomisations create six groups (Fig. 1). Both randomisations are in blocks of varying size and stratified by hospital Trust and specialty (abdominal surgery/obstetric surgery). The sequences of random allocations were generated by computer in advance of starting to recruit. Local research team members access a participant’s allocation via the Internet. The allocation is concealed until a participant’s eligibility and consent have been documented and information to identify the participant uniquely has been entered.

At the beginning of the operation, a member of the local research team (trainee or consultant surgeon/research midwife or nurse) logs on to the randomisation website (within the study database) using a secure password-protected computer system and enters the information needed to proceed to randomise the participant. Depending on the randomisation result, the dressing group allocation is disclosed immediately or the user is advised to log back into the website after the wound has been closed and enter the time of wound closure, after which the allocation is disclosed.

The second randomisation, the disclosure of allocation before or after wound closure, relates to objective 2. During preliminary discussions about the trial, the trials unit proposed that randomisation should occur after wound closure, to prevent surgeons closing the wound in different ways depending on the allocation, but surgeons considered that this would be problematic. The second randomisation allows the trial to test the feasibility of randomising after wound closure to be tested. Moreover, if photographs of the closed wound can be obtained in the operating theatre and subsequently assessed for the quality of wound closure, the effect of timing of randomisation on wound closure can be investigated.

### Blinding

It is not possible to blind surgeons, participants or staff caring for participants to the dressing allocation. However, we plan to blind research staff assessing outcomes 4–8 weeks after randomisation and methods to achieve blinding are being piloted to test their feasibility and acceptability for the main trial. These include requiring the reference SSI assessment and the WHQ to be completed by healthcare professionals who have not been involved in a participant’s care during the index admission. (The study also requires these assessments to be done by different people, in order to validate the WHQ.) The success of blinding among assessors of SSI 4–8 weeks after randomisation (healthcare professionals completing the reference SSI assessment or the WHQ) is being assessed by asking them which study group they think that the participant is allocated to.

If the occurrence of an SSI can be assessed from a photograph after a dressing has been removed, the assessment of the photograph could be blinded. For this reason, the trial is testing the feasibility of participants submitting wound selfies securely to the trial database.

### Integrated qualitative research

A qualitative study is integrated into the pilot study, to provide insights into the feasibility and potential design of a main trial. The study uses semi-structured interviews to explore participants’ experiences and the acceptability of participating in the trial, staff experiences of delivering trial processes/follow-up, and participant/staff perspectives on reasons for protocol deviations. All patients who agree to participate in the pilot RCT are also asked if they are willing for their contact details to be shared with the qualitative research team. Patients who provide written consent to be contacted form the sampling frame for the interviews. Potential interviewees are purposefully sampled from this frame, with the intention of including a range of individuals based on the following criteria:

- surgical specialty (upper GI, lower GI, or obstetric);
- surgical approach (laparoscopic or open procedures);
- nature of hospital admission (elective or unplanned);
- Bluebelle study allocation (simple dressing, ‘no dressing’ or ‘glue-as-a-dressing’).

Healthcare professionals considered to be ‘key informants’ are purposefully selected for potential interview. ‘Key informants’ are defined as any individual with responsibility for caring for Bluebelle participants or delivering aspects of the pilot RCT (e.g. recruitment, randomisation, follow-up).

Topic guides have been developed in the light of earlier findings (Phase A [16]) and are being iteratively developed in light of emerging findings as data collection and analysis proceeds. Interviews will be conducted face-to-face or via telephone by members of the qualitative research team (LR and CM).

Interviews with participants explore the acceptability of trial interventions and trial processes, reasons for withdrawal (if appropriate) and sources of non-adherence (i.e. instigated by staff or participants themselves). Participants may be interviewed at two time points: soon after surgery (within a week); and around their 4–8 week follow-up visit/assessment. A major finding from the qualitative research during Phase A was that clinical staff anticipated challenges in delivering the trial in practice. Therefore, interviews with healthcare professionals are exploring their experiences of implementing study protocols in practice and perceived reasons for non-adherence.

### Trial interventions

Participants are randomised to one of three standard dressing groups: simple dressing (the comparator, believed to represent usual care [17]), no dressing or glue-as-a-dressing.

A simple dressing is defined as a covering (opaque or transparent) that is applied directly to an already closed wound, over the entirety of the wound, adherent around its entire perimeter or surface in contact with the skin. It may or may not have absorbent properties. Table 1 shows examples of commonly available simple dressings. Hospital Trusts may have other types available and use the dressing that represents usual care.

**Table 1:** Examples of commonly used simple dressings

Bioclusive®	
C-View®	
Hydrofilm®	
Opsite®	
Mepore®	
Tegaderm®	

Tissue adhesives are topical skin adhesives. In this trial, they are applied according to the manufacturers’ instructions and must be applied only to the surface of an already closed primary wound, acting as a dressing (not to close the wound, i.e. below skin level). A recent survey found that glue-as-a-dressing is a dressing strategy that is often used by general surgeons [17] but it is considered here as one of the two interventions, compared with a simple dressing (the comparator). Table 2 shows examples of commonly available tissue adhesives that can be used.

**Table 2:** Examples of commonly used types of tissue adhesive

Dermabond ProPen®	
Epiglu®	
Histoacryl®	
LiquiBand®	

In the no dressing group, at the end of the operation when the skin has been closed, no simple dressing or tissue adhesive is applied to the wound. The wound is therefore left exposed without a covering as is the standard approach for many types of surgery, particularly in children [15].

The following aspects of wound care apply to all interventions:

- a participant’s wounds should be dressed according to the participant’s treatment allocation throughout their hospital stay;
- when a participant has more than one wound (e.g. multiple port sites for laparoscopic surgery), all the eligible wounds are dressed according to the treatment allocation;
- re-dressing of a wound in hospital may be needed if there is slow discharge or ongoing seepage of fluid (‘ooze’) from the wound in the first 24 h or if a SSI occurs (i.e. after the outcome of interest has been ascertained). For the former, a simple gauze swab can be applied to the area that is oozing; this is allowed in the no dressing group as well as the other groups. The swab should be taped in place temporarily and not around its entire perimeter. Examples of swabs that can be used are listed in Table 3. If oozing continues, the clinical team may apply any dressing to the wound (or re-suture it if necessary); where this represents deviation from the allocated dressing, the action is documented in the case report forms (CRFs);

**Table 3:** Example of swabs that can be used to manage wound exudate

Gauze swabs	
Filmated gauze swabs	
Non-woven fabric swabs	
Filmated non-woven fabric swabs	
Knitted viscose	
Paraffin gauze dressings
- Co-interventions that may influence SSI rates (e.g. the use of prophylactic antibiotics and other aspects of pre-, peri- or postoperative care) will be allowed at the discretion of the team and hospital looking after the participants. Their distribution by allocated group is being monitored, in view of the risk of bias due to differential implementation of co-interventions when the usual care team is not blinded to the allocated dressing. This information will inform decisions about the need to standardise care when designing the main trial;
- To encourage adherence to treatment allocation, colour-coded skin transfers showing the study logo and the dressing allocation are provided. These are applied on the participant’s skin, near to the surgical wound(s), as a reminder to healthcare professionals looking after the patient about the patient’s participation in the trial and the allocated dressing strategy (Fig. 3). The skin transfer disappears over time and is not visible by the time of the 4–8-week assessment.

**Fig. 3:**
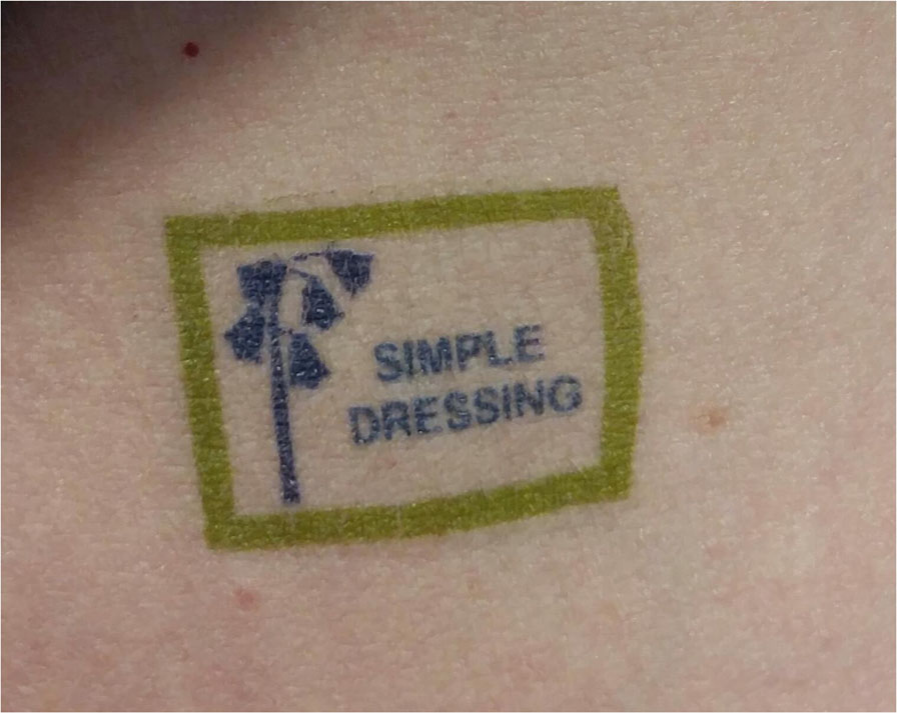
Skin transfers. Image shows a skin transfer applied near to the wound(s) to promote adherence to the randomised dressing allocation

### Study centres and surgeons

Four NHS Hospital Trusts are taking part, one of which is recruiting participants having either abdominal surgery or Caesarean section. (Three Trusts had agreed to take part at the outset and a fourth Trust, recruiting patients having abdominal surgery, joined in month 6.) All general surgical teams carry out a wide range of operations. The participating obstetric unit carries out about 1800 Caesarean sections each year. Since the operations are all being carried out as part of the usual care of participants, there are no restrictions on operating personnel or ward care.

### Primary outcome

The primary outcome is successful screening of a participant, determination of the participant’s eligibility and consent to be randomised in the pilot trial, at the time of randomisation. This information, together with the denominator describing the total number of participants approached, will establish whether recruitment into the main trial is possible.

### Secondary outcomes

These will include:

1. Adherence to disclosure of dressing category allocation at the designated time
2. Adherence to the allocated dressing type by the usual care team during the index hospital admission and, if applicable, reason for non-adherence
3. Quality and completeness of the data for different outcomes anticipated to be measured in the main trial (see below; assessed at different times), including component assessments contributing to an overall judgement about the occurrence of a SSI
4. Adherence to the follow-up schedule
5. Documentation of co-interventions (e.g. details of hair removal, use of skin cleansing agents, type of wound closure methods, prescription of prophylactic antibiotics) and classification of surgery as ‘clean’, ‘contaminated’ or ‘dirty’ at the time of the operation
6. Completion of the reference SSI assessment at 4–8 weeks by a blinded observer
7. Completion of a wound healing questionnaire (WHQ [18] at 4–8 weeks by a blinded observer and by the participant
8. Completion of a wound management questionnaire (WMQ; developed during Phase A) up to 4 days after randomisation by an observer or the participant (if discharged early, e.g. day-case surgery)
9. Completion of a wound experience questionnaire (WEQ; developed during Phase A) up to 4 days after randomisation by the participant
10. Assessment of wounds from digital photographs at 4–8 weeks, if submitted

Bluebelle is a feasibility study and some of these secondary outcomes were developed in Phase A and are being piloted in Phase B. Therefore, detailed scoring methods are not currently available. We will report the questionnaire response rates, the number of fully completed questionnaires and rates of missing items in this pilot RCT as part of the evaluation of the acceptability of the new questionnaires to patients. The data from the pilot trial are also being used to validate some of the questionnaires.

### Anticipated outcomes in a subsequent main trial

The following outcomes are expected to be assessed in a main trial; the ability to collect these outcomes is, therefore, a key focus of the pilot trial:

1. Occurrence of a SSI up to 4–8 weeks after randomisation (primary)
2. Wound management questionnaire
3. Patient reported outcomes: wound experience questionnaire, documenting symptoms and experiences of the wound and dressings, and generic health status, assessed by the EQ-5D-5L [22]
4. Wound complications arising up to 4–8 weeks after randomisation
5. Resource use up to 4–8 weeks, including length of postoperative hospital stay, rates of re-admission and duration

### Trial procedures and data collection up to four days after randomisation

The schedule of data collection is described in the SPIRIT Figure (Fig. 2).

Surgery and closure of wounds takes place as per usual practice. A member of theatre personnel, or research team, records: (1) the time taken to close the wound; (2) use of laparoscopic or open surgical methods; and (3) the method of wound closure. A member of theatre personnel or research team may also take one or more digital photographs of the wound(s). If randomised to disclosure of allocation after wound closure, the time of completing wound closure must be entered into the database to obtain the allocation. A range of methods are being used to promote adherence to the randomised dressing allocations in hospital, adapted to the circumstances of participating hospitals. These include simple labels, to be attached to medical notes or placed by the bedside, as well as skin transfers (Fig. 3).

Up to four days after surgery, after any early wound care that is required, a healthcare professional completes the WMQ. This questionnaire captures information about aspects of the participant’s wound management. If discharged early, e.g. after day-case surgery or the day after surgery, the research team gives the participant the WMQ to complete by day 4 and send back to the trials unit in a pre-paid envelope.

The local research team gives each participant the WEQ to complete up to four days after surgery. For participants who are in hospital at this time, the WEQ is collected by the research team. If discharged early, e.g. after day-case surgery or the day after surgery, the participant completes the WEQ at home by day 4 and sends it back to the trials unit in a pre-paid envelope.

### Data collection up to 4–8 weeks after randomisation

Local research teams give each participant a copy of the EQ-5D-5L and a pre-paid reply envelope to take home and instruct them to complete and return it if a wound becomes infected or problematic. The reason for asking for the EQ-5D-5L to be completed is to try to document the peak impact of a wound problem on health status to inform a future health economic evaluation.

Several follow-up assessments are required 4–8 weeks after randomisation. First, the participant needs to complete a copy of the EQ-5D-5L and the WHQ; these questionnaires can be given to participants at discharge from hospital or posted shortly before they fall due. Around this time, participants who have agreed to send a photograph may also be sent an email or text message including a web-link that allows them to upload a photograph securely. Then, a blinded health professional needs to complete the WHQ; this can be done face-to-face, typically at the same clinic attendance but before the reference SSI assessment, or administered by telephone. Finally, the face-to-face reference SSI assessment is completed by a blinded health professional, who must be different to the one completing the WHQ; this assessment includes eliciting information about potential wound-related complications. Whenever possible, the face-to-face clinic visit is arranged to coincide with a usual care clinic appointment. When this is not possible, travel expenses are offered to the participant. For women who have had a Caesarean section, the WHQ questionnaire is administered by telephone and research midwives then carry out the reference SSI assessment at a home visit.

To promote retention, reminders are sent out to participants who fail to return postal questionnaires promptly. Participants who fail to attend for the face-to-face SSI assessment are offered further appointments.

### Source data

The operation and medical notes, and other information held electronically on hospital information systems, provide source data for participant demographic and baseline information, operation details and postoperative morbidity. The completed participant questionnaires and observer-reported assessments of SSI and wound management are the primary data source for these measures. The data source for secondary adverse outcomes will be the participant’s medical notes. Wound photographs are the primary data source for this outcome.

Staff at participating centres record data on paper CRFs, then use a password-protected web server application to transcribe the data into a custom-designed database, located on a secure NHS server. The database has validation on data fields.

### Assessment and analysis of resource use

The pilot trial will assess the feasibility of collecting healthcare resource use data from the CRFs completed during the admission and at the 4–8 week follow-up. Relevant resource use is expected to fall under the following categories:

1. The wound dressing itself
2. Postoperative resources expended in the hospital setting
3. SSI-related care
4. Post-discharge resources expended in primary care

The analysis will involve an assessment of the quality and completeness of the data for each data item, for example data on type of wound dressing and frequency of use, and other data on healthcare services provided in the hospital setting (i.e. post-randomisation hospital stay and follow-up outpatient appointments to assess wound healing). It will also start to consider the main drivers of cost, so that these can be accurately collected within the main trial.

Unit cost information associated with different types of care will be collected or estimated in preparation for the main trial and to ensure that these costs are available or can be generated. Particular attention will be paid to understanding the costs associated with SSIs as preliminary modelling suggests that these costs, rather than the costs of dressings, are likely to be the main driver of any cost differences between the arms of the trial. Preference-based quality-of-life estimates for the entire cohort will be derived by translating patients’ responses to the EQ-5D-5L over the 4–8 weeks after randomisation into utility values using the latest value set for England [23].

### Trial duration

The trial is timetabled to take up to 11 months, randomising the target number of 330 participants in nine months and then following up the last participants for 4–8 weeks.

### Sample size

A pilot study of 920 eligible participants, will allow a recruitment rate of 36% (corresponding to the target number randomised of 330) to be estimated with a 95% confidence interval (CI) of 32–39, and a recruitment rate of 60% with 95% CI of 56–64. For the simple dressings group, we anticipate an adherence rate of 90%. Assuming a 36% recruitment rate and 110 randomised participants per group, a 90% adherence rate will be estimated with a 95% CI of 82–95. We have no information on which to base any estimate of adherence in the no dressing and glue-as-a-dressing groups. However, if adherence were less than 70% in either group, we would conclude that randomisation to the group in question in the main trial would not be feasible.

### Statistical analyses

Summary descriptive statistics to inform plans for the main trial will be reported including:

1. The number of potentially eligible participants per month per centre
2. The percentage of these potential participants confirmed as eligible
3. The percentage of participants agreeing to be randomly allocated to dressing type in the pilot RCT
4. The percentage of randomised participants receiving the allocated treatment and completing outcome measurements at the 4–8-week assessment
5. Rate of, and reasons for, non-adherence to allocation at both a wound and participant level
6. Mean number of wounds per participant
7. Mean (or median if skewed) time from wound closure to randomisation
8. Mean (or median if skewed) time to complete randomisation process
9. Completeness of data items and reasons for missing data
10. Rate of unblinding of outcome assessors and reasons for unblinding
11. Secondary outcome measures relating to wound closure will be compared between groups, if the data allow

Only the statisticians will have access to the data. The analysis population will include all randomised participants. Results will be described by centre and by specialty as well as overall. If the data allow, subgroup analyses of the secondary outcome measures relating to wound closure will estimate the interaction of timing of randomisation by dressing group.

The primary analysis will take place when follow-up is complete for all recruited participants. During the pilot trial, we are monitoring recruitment and adherence periodically. The study steering committee will review safety data. In any interim reports, for example about withdrawals after randomization, the data will be presented by group with uninformative labels to keep the allocation masked.

### Dissemination

The results of the pilot trial will be reported in peer-reviewed journals, in a report to the funder and as a lay summary to participants. We will apply authorship criteria established by the International Committee of Medical Journal Editors.

### Changes to the protocol since first approved

This paper describes protocol version 5 (21 October 2016). Two substantial amendments have been approved.

Version 2 was the first approved protocol. The first amendment (from v2 to v3) was approved (8 December 2015) before starting to recruit. Due to the short duration of the pilot trial, REC approval was obtained for a design as conceived at the outset for the study, before Phase A was completed. The first amendment described revisions to this protocol that were required as a result of the findings from Phase A [16]. The funding application for the study always envisaged that Phase B would be informed by Phase A. The time constraints for the overall study (both Phase A and Phase B), combined with the time needed to obtain REC approval, made it inevitable that an amendment would be required after Phase A ended. This amendment included: changing the trial population to include patients having unplanned operations; substituting the intervention of a complex dressing with glue-as-a-dressing; and addition of the WMQ and WEQ as secondary outcomes.

The need for a second amendment (v3 to v4, then to v5 to accommodate a change requested by the REC) arose from initial difficulties in recruiting patients having unplanned surgery and obtaining patient-reported follow-up questionnaires. This amendment (approved 6 September 2016) substituted a 4-h period between giving the PIL to a potential participant and requesting consent by the statement ‘as long as possible’, qualifying this as ‘usually more than 4 hours for elective surgery and usually more than 1 hour for unplanned surgery’. We justified this on the basis of feedback from trainee surgeons and research nurses that potential participants did not require ‘thinking time’ in order to decide whether to take part. We also revised the protocol to allow the 4–8-week WHQ to be administered over the telephone by a health professional, as well as face-to-face. We applied for the trial to be adopted by the Health Research Authority shortly after submitting the amendment, in order to obtain approval for another NHS Trust to recruit patients having abdominal surgery.

## Discussion

The short duration of the pilot trial, constrained by the length of the overall research contract, has been a challenge. The design of pilot trial depended on the findings of Phase A and the design was finalised shortly before starting to recruit. Despite this, final preparation and the two site initiation visits took place in just ten weeks, after notification that the first amendment had been approved. Development of the trial database was split into two releases, one to allow recruitment and randomisation, and the second for entry of data captured on paper CRFs.

### Status of the pilot trial

Recruitment is ongoing and is on target to achieve at the pre-specified sample size in the scheduled time. The interventions appear to be acceptable to potential participants and over 50% of those who have been approached to take part have given written consent. After some initial deviations, adherence has also been good; in particular, members of local research teams have reported that the skin transfers are an acceptable and effective way to promote adherence. Qualitative interviews have also indicated that the interventions are acceptable and that there have been few issues with adherence. Further details about the feasibility outcomes will be reported fully in due course.

## Additional files

Additional file 1: SPIRIT 2013 Checklist: Recommended items to address in a clinical trial protocol. (PDF 132 kb)

Additional file 2: Participant Information Leaflet. (PDF 273 kb)

Additional file 3: Participant Consent Form. (PDF 30 kb)

## Abbreviations

CI: Confidence interval
CONSORT: Consolidated standards of reporting trials
CRF: Case report form
EQ-5D-5L: 5-level EuroQol health status questionnaire
HTA: Health Technology Assessment
NHS: National Health Service
NIHR: National Institute for Health Research
PIL: Patient information leaflet
RCT: Randomised controlled trial
REC: Research Ethics Committee
SMG: Study Management Group
SSC: Study Steering Committee
SSI: Surgical site infection
UK: United Kingdom
WEQ: Wound experience questionnaire
WHQ: Wound healing questionnaire
WMQ: Wound management questionnaire
