# Supplementary material for: A mixed-methods feasibility and external pilot study to inform a large pragmatic randomised controlled trial of the effects of surgical wound dressing strategies on surgical site infections (Bluebelle Phase B): study protocol for a randomised controlled trial
Source: Trials. 2017 Aug 29;18:401. doi: 10.1186/s13063-017-2102-5 (PMC5576037; doi:10.1186/s13063-017-2102-5)
Supplement: Supplementary file 2 — Participant Information Leaflet. (PDF 273 kb) [file 13063_2017_2102_MOESM2_ESM.pdf]

The Bluebelle Study:
A feasibility study of three wound dressing strategies in elective and unplanned surgery

Participant Information Leaflet

<Trust logo>

Local Principal Investigator:
<Insert PI name and contact details>

Local contact details:
<Insert coordinator/clinician name and contact details>

Trust study number (local site): <xxxxx>
HTA reference (if applicable):12/200/04
IRAS ID: 163427

Contact details

The Bluebelle Study Research Team
<Insert local team contact details>
Address:
Tel:
Fax:
Email: bluebelle-study@bristol.ac.uk

Contents

What is the purpose of the study? 4
Why have I been invited? 4
What will happen to me if I take part? 4
What will I have to do? 5
What alternatives are there to taking part in the study? 6
What are the possible disadvantages and risks of taking part? 6
What are the possible benefits of taking part? 7
What happens when the research study stops? 7
What if there is a problem? 7
Will my taking part in the study be kept confidential? 8
What if relevant new information becomes available? 8
What will happen if I don’t want to carry on with the study? 8
What will happen to the results of the research study? 9
Who is organising and funding the research? 9
Who has looked at the study? 9
Further information 9

You are being invited to take part in a research study. Before you decide whether to take part or not, you need to understand why the research is being done, and what it would involve for you. Taking part in research is voluntary; it is up to you to decide to join the study. You are free to withdraw at any time and, if you choose not to take part or to withdraw from the research, you do not have to give any reason for your decision. Nobody will be upset and the standard of care you receive will not be affected if you decide not to take part.

Please take time to read the following information carefully. **One of our team will go through the information leaflet with you, explain the study in more detail and answer any questions you have**. If anything is not clear or you would like more information, do not hesitate to ask a member of the local research team (see contact details on page 2). Talk to others about the study if you wish, such as friends or relatives, and take time to decide. If you would like to take part, you will be asked to confirm this by signing a separate consent form. You will be given a copy of the form for your records.

What is the purpose of the study?
The Bluebelle study is a small study to find out if a larger study that compares different types of wound dressings can be done. The larger study will provide high quality information about the best way to dress surgical wounds. It will investigate the use of wound dressings following common operations involving the stomach/tummy and including Caesarean section.

A wound dressing is a product placed over a wound that has already been closed (e.g. with stitches or clips). There are many different types of wound dressings that can be used following common operations. Most are simple and inexpensive, not unlike plasters used in the home but larger. Alternatively, the surgeon can spread a transparent film called tissue adhesive along the length of the wound, in this study we refer to this as this glue-as-a-dressing. A third possibility is for no dressing to be used at all. This is quite common for certain operations and particularly for children who have surgery.

In this study, we are inviting patients and women having their baby by Caesarean section to be assigned to have simple dressings, glue-as-a-dressing or no dressing on their wounds. Our primary aims are to find out whether assignment to one of these options is acceptable and whether staff use the assigned type of dressing, i.e. avoid reverting to the way they usually dress wounds. This is important so that the research team can judge whether it is possible to conduct a full scale study.

Why have I been invited?
You have been invited to take part because you are scheduled to have one of the operations that is being considered for inclusion in the Bluebelle pilot study.

What will happen to me if I take part?
A member of the research team will come and speak to you on the day of your operation or within a few days before hand. This will give you the opportunity to ask any questions and have a further explanation as to what the study entails.

If you are interested in participating you will be asked to give your written consent to take part in the study before your operation and you will be given a copy of the consent form and this information leaflet to keep.

In this study a third of the participants who take part will have a simple dressing, a third will have glue-as-a-dressing and the other third will have no dressing applied to their wound at the end of surgery. Simple dressings are ones that cover the wound and stick to the skin around the wound. Glue-as-a-dressing is applied along the length of a wound, after the edges have been closed, e.g. by stitches; it is transparent and seals the surface of the wound. For participants having no dressing, a piece of cloth or gauze may be used to soak up any fluid from the wound, but the wound will not be completely covered. It is important to remember that wounds will still be secure and intact, even if there is no dressing placed over the top of the wound. Dressing a wound does not affect the risk of the wound edges separating. A member of the theatre team may photograph your wound in theatre, with your consent. No one will be able to identify you from the photograph(s) which will be taken on a hospital approved device.

At the moment, nobody knows whether it is better to have a simple dressing, glue-as-a-dressing, or no dressing. A large trial comparing these ways of dressing a wound would need to ensure that participants assigned to different groups are similar. The best way to achieve this is to assign participants by chance: a process called randomisation. We want to test whether this method of allocation is acceptable to participants. This means that you will have an equal chance of having a simple dressing, glue-as-a-dressing or no dressing.

After the operation, you may have a small sticky plaster, or a skin transfer (like a children's skin 'party sticker') close to the wound to remind the staff that you are participating in the study. If you don't want anything like this, you can still take part – just tell the person asking for your consent.

If you are allocated to receive a simple dressing or glue-as-a-dressing, the dressing type you are allocated to will be used to dress your wound throughout your hospital stay. Regardless of which group you are allocated to, a study clinician will assess your wound during your hospital stay (this will include them completing a wound management questionnaire) and assess your wound at a 4-8 week post-surgery follow-up clinic appointment. The researchers reviewing your wound at the 4-8 week follow-up appointment will not know which dressing you were given after your operation, and therefore we would ask you not to tell them so that they can look at your wound without knowing this information. At the 4-8 week follow-up clinic the study clinician may take photographs of your wound as part of their assessment. Participants who have had a Caesarean section who are unable to attend the hospital visit may be offered a home visit and a separate wound assessment over the telephone. Participants who attend the clinic for their follow-up appointment may also have a separate wound assessment over the telephone if it is not possible to do this assessment during the clinic visit.

What will I have to do?

We will ask you to complete three different questionnaires during this study: a quality of life questionnaire, a wound experience questionnaire and a wound healing questionnaire. In addition to these, if you are having your surgery as a day case or you are discharged

before a healthcare professional has completed a wound management questionnaire, you will be asked to complete this questionnaire too up to 4 days after your surgery. These questionnaires will take around 10-15 minutes each to complete, and may be posted, completed electronically or completed during your hospital stay or at a clinic visit.

The quality of life questionnaire asks questions about how you feel, what activities you can do and your quality of life. We will ask you to complete this before your operation (pre-op), and at 15 days and 4-8 weeks after your operation. We will also ask you to complete this questionnaire if, at any time, you have a problem with your wound. We would like you to consider taking a photograph of your wound and sending it to us when you complete the 4-8 week post-op quality of life questionnaire, and again if you complete the questionnaire at any other time (if you have a problem with your wound). We will give you instructions which will explain how to do this. Completion of follow-up questionnaires and/or sending wound photographs will automatically enter you into a prize-draw.

The wound experience questionnaire asks questions about wound comfort and the dressing (or not having a dressing) and we will ask you to complete this up to 4 days after your operation.

The wound healing questionnaire focuses specifically on your wound and how it has healed. We will ask you to complete this 4-8 weeks after your operation.

You will be asked to return to have your wound assessed 4-8 weeks after your operation. For some participants, this will be at the same time as a follow-up appointment with the surgical team. For others, the appointment will only be to have the wound assessed. In addition to your 4-8 week follow-up visit, we may also telephone you to complete a wound assessment over the phone.

You may also be asked if you are happy to take part in a further interview about your experiences of being in the trial. Separate information (and a consent form if appropriate) would be provided for you to consider.

Any interviews that you take part in and/or study photographs will become part of the study data set. These data will be stored on a secure database at the co-ordinating centre in Bristol.

Expenses
Travel expenses will be available for the 4-8 week follow-up visit to hospital if the visit is for research purposes only, and not a routine visit.

What alternatives are there to taking part in the study?
If you decide not to take part in the research study, then your wound will be dressed using the standard types of dressings available at this hospital.

What are the possible disadvantages and risks of taking part?
There are examples of medical practice where doctors currently apply wound dressings, and examples where they do not. We therefore do not anticipate any risks. If a wound problem occurs (e.g. infection or leakage) it will be treated in the normal way.

Before participating you should consider if this will affect any insurance you have and seek advice if necessary.

What are the possible benefits of taking part?
The information we will get from the study will be very helpful to the NHS and to future patients, and women having Caesarean sections, who need operations which carry a risk of wound infection that might be influenced by the use of wound dressings.

What happens when the research study stops?
At the end of the research study, or if you withdraw from the research study for any reason, you will continue to be treated and followed up in the normal way.

What if there is a problem?
If you have any concerns or questions about this study, please contact the research team listed on the inside front cover of this leaflet. Alternatively you can discuss these with a member of the research team who will come to see you before your operation. Please feel free to ask any further questions before deciding to take part in the trial, or at any time during the study.

If you have concerns about the way you have been approached or treated during the course of the study, you may wish to contact the Patient Advice and Liaison Service (PALS) on:

<Insert local details>
Address:
Tel:
Text phone:
Email:

If you wish to make a formal complaint, please write to:

<Insert Chief Executive address for site>

Or telephone the Patient Complaints Manager on

<Insert telephone number>

We have no reason to believe that you will be placed at any greater risk by taking part in this research study. However, if something goes wrong and you are harmed during the research study there are no special compensation arrangements. The University Hospitals Bristol NHS Foundation Trust cannot offer no-fault compensation and is unable to agree in advance to pay compensation for non-negligent harm. Ex-gratia payments may be considered in the case of a claim.
If anything goes wrong as a consequence of taking part in the trial because negligence has occurred, University Hospitals Bristol NHS Trust, who is sponsoring the trial, will compensate you. Negligence would include, for example, a situation in which injury is caused by a deviation from the study protocol by the researcher. Your legal right to claim compensation for injury where you can prove negligence is not affected. If you are harmed and this is due to someone's negligence then you may have grounds for a legal action against the University Hospitals Bristol NHS Trust, but you may have to pay your legal

costs. The normal National Health Service complaints mechanisms will still be available to you.

Will my taking part in the study be kept confidential?
All information which is collected about you during the course of the research will be kept strictly confidential. The information that will be collected includes personal information such as your name, address and NHS number, to allow us to keep in touch with you during your participation in the research, and information about your health, to allow us to compare the three dressing types. The information collected will be stored securely in paper format at the hospital and on a secure database at the co-ordinating centre in Bristol. All information will be accessed only by authorised members of staff involved in the research including the hospital research team and staff at the coordinating centre who are managing the research and who will be responsible for aspects of your follow-up.

Your medical notes will need to be seen by authorised members of the hospital research team so they can collect information needed for this research study. With your consent, your GP will also be informed that you are taking part in the research study. Your GP may be asked to provide information from your records which is required for the research. Occasionally, other members of NHS staff or research staff may need to check your medical records. This will be done by NHS staff or by researchers who are bound by the same rules of confidentiality as all NHS staff. The confidentiality of your medical records will be respected at all times. Under no circumstances will you be identified in any way in any report arising from the study.

With your consent, after you leave the study we would still like to know how you are progressing using information collected routinely in the NHS and information held routinely by the NHS Information Centre. Information will be obtained from the NHS Information centre's 'Medical Research Information Service'. Any information received in this way remains confidential and will only be used for the purpose of this research.

What if relevant new information becomes available?
Sometimes we get new information about the treatment being studied. If this happens, your research doctor will tell you and discuss whether you should continue in the study. If you decide not to carry on, your research doctor will make arrangements for your care to continue. If you decide to continue in the study, your research doctor may ask you to sign an agreement outlining the discussion.

What will happen if I don't want to carry on with the study?
If you want to withdraw from the research, or your doctor decides it is in your best interests for you to withdraw for any reason, you are free to do so at any time and your care will continue in the usual way. If you withdraw before your operation, then doctors will use the standard dressing type at your hospital. If you withdraw after your operation but before you have completed the post-operative surgical site infection assessments and quality of life questionnaire then you will no longer be asked to do these things.

If, after the operation, you do not want to take part any further, we will ask you to tell us what you would like us to do with any information that have already been collected. Information collected up to the point at which you decide to withdraw is still valuable for the study. Therefore, we will ask whether you want the information destroyed, or whether we

can use the information but without having any further contact with you. We will also ask whether we can continue to follow your progress using standard NHS records, without contacting you.

What will happen to the results of the research study?
The results of the research will not be known until sometime after the last participant has entered the study (about 2 years after the start of the study). The results may be reported in medical journals or presented at meetings but your identity will not be disclosed. During the course of the study we will ask you if you would like to receive a summary of the results by post after the research has finished.

Who is organising and funding the research?
The research is funded by the National Institute for Health Research - Health Technology Assessment Programme (12/200/04). The views expressed are those of the author(s) and not necessarily those of the NHS, the NIHR or the Department of Health. The University Hospitals Bristol NHS Foundation Trust has overall responsibility for conduct of the study. The research is being organised and run on their behalf by the Clinical Trials and Evaluation Unit, University of Bristol.

Who has looked at the study?
All research in the NHS is looked at by an independent group of people, called a Research Ethics Committee, to protect your safety, rights, wellbeing and dignity. This study has been reviewed and given a favourable opinion by NRES Committee South West – Frenchay REC.

Further information
You can obtain **general information on clinical research** from the UK Clinical Research Collaboration (UKCRC) who produce a booklet called "Understanding Clinical Trials". This provides in-depth information on the design and conduct of clinical trials and aims to answer the questions of those considering taking part.

Electronic copies can be downloaded from the UKCRC website:
www.ukcrc.org/publications/informationbooklets.aspx.

Printed copies can be requested by emailing:
info@ukcrn.org.uk

Or contacting:
UK Clinical Research Collaboration,
20 Park Crescent,
London, W1B 1AL
Tel: 020 7670 5452

General information about research can also be found at www.uhbristol.nhs.uk/research or there are leaflets available if you would like to be sent one. Please ask.

Funded by NHS National Institute for Health Research

Thank you for taking the time to read this leaflet.
