# Supplementary material for: A mixed-methods feasibility and external pilot study to inform a large pragmatic randomised controlled trial of the effects of surgical wound dressing strategies on surgical site infections (Bluebelle Phase B): study protocol for a randomised controlled trial
Source: Trials. 2017 Aug 29;18:401. doi: 10.1186/s13063-017-2102-5 (PMC5576037; doi:10.1186/s13063-017-2102-5)
Supplement: Supplementary file 3 — Participant Consent Form. (PDF 30 kb) [file 13063_2017_2102_MOESM3_ESM.pdf]

Trust study number (local site):
HTA reference (if applicable): 12/200/04
IRAS ID: 163427

<<Insert PI name>>
<<Insert PI address>>
<<Insert PI tel number>>
<<Insert PI fax number>>

Trust logo or replace with <*To be printed on site headed paper*>

Participant Study ID [ ]

The Bluebelle Study: a feasibility study of three wound dressing strategies in elective and unplanned surgery

Participant Consent Form

Please ask the participant to complete the following: ***Participant to initial***

		**Initials**
1.	I confirm that I have read and understood the Participant Information Leaflet (Pilot RCT, dated ____/____/____, version _____).	
2.	I have had the opportunity to ask questions about the study and received satisfactory answers to my questions.	
3.	I understand that I am free to withdraw from the study at any time without giving a reason and that withdrawing from the study will not affect my medical care or legal rights.	
4.	I give permission for sections of my medical records to be looked at by the study team, the regulatory authorities or the hospital trust overseeing the research. I understand that strict confidentiality will be maintained.	
5.	I agree to my GP being informed of my participation in this study.	
6.	I give permission for my personal data (including any photographs) to be stored for the duration of the study.	

		Yes	***No***	***Initials***
7.	I agree to take part in this study which may include a clinician taking photographs of my wound(s) (if you prefer not to have photographs taken, your participation in the rest of the study will not be affected).			
8.	I agree to take part in this study which may include me taking and sending photographs of my wound(s) to the study team (if you prefer not to take photographs yourself, your participation in the rest of the study will not be affected).			
9.	I give permission for a skin transfer to be applied after my operation to remind staff that I am participating in the study.			
10.	Do you agree to your contact details being sent to a researcher at the University of Bristol/Birmingham so you can be contacted about the possibility of interviews regarding your experiences in the Bluebelle pilot trial?			
11.	I agree to take part in this study.			

Name of participant	Signature	Date
Name of person taking consent	Signature	Date

1 copy for participant; 1 for research team (original); 1 to be kept with hospital notes

The research is funded by the National Institute for Health Research Health Technology Assessment Programme (12/200/04). The views expressed are those of the author(s) and not necessarily those of the NHS, the NIHR or the Department of Health.
